# Association of physical activity and mental health symptoms to the academic performance of physical therapy students in Saudi Arabia: A cross-sectional study

**DOI:** 10.1097/MD.0000000000046934

**Published:** 2026-01-02

**Authors:** Abdullah Ibrahim Alhusayni

**Affiliations:** a Department of Health Rehabilitation, College of Applied Medical Sciences at Shaqra, Shaqra University, Shaqra, Saudi Arabia.

**Keywords:** academic performance, anxiety, depression, physical activity, Saudi Arabia

## Abstract

Physical activity and mental health may influence the academic performance. The aim of this study was to assess the association between physical activity, anxiety, depressive symptoms, and academic performance among physical therapy students in Saudi Arabia. A cross-sectional survey was conducted with 600 undergraduate physical therapy students. Participants completed questionnaires including Physical Activity Rating Scale (PARS-3), Generalized Anxiety Disorder 7-item scale (GAD-7), Patient Health Questionnaire-9 (PHQ-9), and grade point average (GPA). Multivariate regression analysis was conducted to find independent predictors of academic performance. Most participants were male (72.16%), aged from18 to 22 years (89.83%), and had low physical activity levels (88.83%). Mean ± SD scores were 11.16 ± 7.41 for PARS-3, 4.5 ± 4.30 for GAD-7, and 6.87 ± 4.81 for PHQ-9. Mild or greater anxiety affected 42.3%, and 63.2% reported mild or more serious depressive symptoms. No significant associations were observed between PARS-3, GAD-7, or PHQ-9 categories and GPA. Regression analysis indicated that being older was associated with lower odds of high academic performance, while living with one’s family significantly increased the odds of achieving a high GPA. Despite the high prevalence of low physical activity and psychological distress, no significant associations with academic performance were found. Given the association of age and living with one’s family, the importance of considering demographic and social-context factors is underscored when developing strategies to support student success.

## 1. Introduction

Academic performance is a key indicator of the success of a student and future professional competence, reflecting not only their mastery of knowledge but also their ability to translate learning into clinical practice.^[1]^ In the context of health sciences and medical students, academic achievement has implications that extend beyond grades, as it directly influences the development of critical thinking, problem-solving skills, and readiness to deliver quality patient care, collectively affecting the health condition of nation in the future.^[2]^ Understanding the factors that shape academic outcomes is therefore essential for educators and policymakers.

Physical activity is a cornerstone of health promotion and disease prevention, with well-documented benefits for cardiovascular health, musculoskeletal fitness, mental well-being, and overall quality of life.^[3–5]^ In university populations, engaging in regular physical activity has also been associated with improved cognitive functioning, stress reduction, and better academic outcomes.^[6]^ However, despite these established benefits, many university students exhibit insufficient levels of physical activity, particularly in regions undergoing rapid sociocultural changes, such as Saudi Arabia.^[7–9]^

University life is often characterized by increased academic demands, social pressures, and lifestyle transitions, which can negatively impact on students’ mental health.^[10]^ Anxiety and depression are prevalent among university students worldwide,^[11]^ including in Saudi Arabia,^[12]^ where mental health stigma and limited access to psychological services may exacerbate the burden of untreated symptoms. Anxiety and depressive symptoms can impair concentration, motivation, and memory, which may in turn adversely affect academic performance.^[13]^ Recognizing these interconnected factors is essential for developing holistic strategies that support students’ well-being and academic success.

Physical therapy students, in particular, face unique stressors related to rigorous academic requirements, clinical training, and professional socialization. Maintaining high levels of academic performance is critical for their progression and future careers. At the same time, their education emphasizes health promotion, making them a key target group for understanding the relationship between lifestyle behaviors and mental health outcomes.^[14]^ Nonetheless, research examining these interrelationships among Saudi Arabian physical therapy students remains scarce.

Given the paucity of local data, there is a need to investigate the associations between physical activity, mental health, and academic performance in this specific student population. Such evidence can guide personalized interventions, inform curriculum design, and support student services that seek to enhance both well-being and educational attainment. Therefore, the primary aim of this study was to assess the association between physical activity, mental health, and academic performance among physical therapy students in Saudi Arabia. These factors were simultaneously evaluated in a cross-sectional framework, which allowed the study to arrive at a comprehensive understanding of how lifestyle and mental health factors relate to academic outcomes in this important health professional cohort.

## 2. Methods

### 2.1. Study design

This was a cross-sectional analytic study, conducted between April 2025-June 2025, to assess the associations between physical activity, mental health symptoms, and academic performance among physical therapy students in Saudi Arabia. The study population comprised undergraduate physical therapy students enrolled at universities across the country. Inclusion criteria required participants to be at least 18 years old and currently enrolled in a recognized physical therapy program in Saudi Arabia. Exclusion criteria included students who were under 18 years of age, not currently enrolled in a physical therapy program, or who submitted incomplete responses.

Ethical approval (Reference Number: ERC_SU_S_202400061) for the study was obtained from the relevant ethics committee at Shaqra University, Saudi Arabia, before data collection commenced. To ensure broad and representative participation, recruitment was carried out through multiple channels, including university mailing lists, student organizations, social media platforms, and direct outreach via faculty coordinators. The survey was distributed online using a secure data collection platform, with availability in both English and Arabic to boost accessibility. Participation was anonymous, and no personal identifiers were collected. Participation was voluntary, and students provided informed consent prior to completing the survey.

### 2.2. Sample size calculation

The required sample size was calculated using the formula $n=\;\frac{Z^{2}\times SD^{2}}{D^{2}}$ to estimate a mean value with a 95% confidence level and specified precision (D), as recommended previously.^[15]^ Assuming a standard deviation (SD) of 0.58, a Z-score of 1.96 (corresponding to 95% confidence), and a desired precision (D) of 0.05, the initial sample size required was approximately 517 participants. To account for anticipated attrition or an incomplete response rate of around 10%, the target sample size was increased to 600 undergraduate physical therapy students.

### 2.3. Data collection and management

Data were collected through a structured, self-administered online questionnaire. The survey comprised several sections. First, students provided demographic and academic information, including age, sex, academic year, and cumulative grade point average (GPA).

Physical activity was assessed using the Physical Activity Rating Scale (PARS-3), a validated 3-item instrument measuring the intensity, duration, and frequency of self-reported physical activity. The questionnaire was revised by Liang *et al* from Wuhan Sports University and its validity and quality have been approved.^[16,17]^ Total score was calculated by multiplying intensity by the sum of duration and frequency (Total score = Intensity × [Duration + Frequency]), with higher scores indicating greater activity levels. Scores were then classified as low (≤19), moderate (20–42), or high (≥43) physical activity.

Anxiety symptoms were measured using the Generalized Anxiety Disorder 7-item scale (GAD-7), a validated screening tool that asked participants how often they experienced specific anxiety symptoms over the previous 2 weeks.^[18]^ Each item was scored from 0 (not at all) to 3 (nearly every day), yielding a total score ranging from 0 to 21. Severity levels were categorized as minimal (0–4), mild (5–9), moderate (10–14), and severe (15–21).

Depressive symptoms were assessed using the Patient Health Questionnaire-9 (PHQ-9).^[19]^ Participants rated 9 depressive symptoms on a scale from 0 (not at all) to 3 (nearly every day) for the previous 2 weeks, producing total scores between 0 and 27. Severity was classified as minimal (0–4), mild (5–9), moderate (10–14), moderately severe (15–19), and severe (20–27).

### 2.4. Statistical analysis

Data were exported securely from the survey platform, checked for completeness and consistency, and stored in a password-protected database. Descriptive statistics were calculated to summarize participant characteristics, physical activity levels, mental health symptoms, and academic performance. Between-group comparisons were performed using GPA categories as the grouping variable, with physical activity (PARS-3), anxiety (GAD-7), and depression (PHQ-9) scores as the dependent variables. Depending on data distribution (as checked by a Kolmogorov-Smirnov test), analysis of variance (ANOVA) or non-parametric Kruskal-Wallis tests with appropriate post hoc comparisons were used for continuous variables, while chi-square tests were employed for categorical variables. A multivariable binary logistic regression was performed with GPA categorized as low (≤3.5 − 4) or high (>3.5 − 4), as the dependent variable. A multivariable logistic regression analysis was conducted to evaluate the independent effects of physical activity, anxiety, and depressive symptoms on academic performance (GPA category), controlling for potential confounders such as age, sex, body mass index (BMI), smoking status, living arrangements, and chronic disease. Adjusted odds ratios (ORs) and 95% confidence intervals (CIs) were reported. Statistical significance was defined as a 2-sided *P* value of < .05. Data analysis and figure designing were conducted using Statistical package for social sciences (SPSS) v.27 software for windows (IBM Corp., Armonk) and GraphPad Prism v.10.3.1 software for windows (GraphPad Software, San Diego), respectively.

## 3. Results

### 3.1. Demographic characteristics

A total of 600 physical therapy students participated in the study (Table 1). The majority of the participants were male (72.16%), while females comprised 27.83% of the sample. Most students (89.83%) were between 18 and 22 years of age, with smaller proportions in the 23 to 27 (5.5%) and 28 to 32 (4.67%) age groups. The vast majority of respondents were Saudi nationals (96.5%), and most reported being single (96.5%).

**Table 1:** Baseline data of the study subjects.

Subjects’ characteristics	Value (N = 600)
Gender	
Male	433 (72.16%)
Female	167 (27.83%)
Age (yr);	
18–22	539 (89.83%)
23–27	33 (5.5%)
28–32	28 (4.67%)
Nationality	
Saudi	579 (96.5%)
Non-Saudi	31 (3.5%)
Marital status	
Single	579 (96.5%)
Married	31 (3.5%)
Smoking status	
Former smoker	47 (7.83%)
Nonsmoker	519 (86.5%)
Smoker	34 (5.67%)
Living arrangement	
Living with family	393 (65.5%)
Not living with family	207 (34.5%)
GPA	
<3	20 (33.33%)
3–3.5	140 (23.33%)
3.5–4	193 (32.16%)
4–4.5	166 (27.66%)
>4.5	81 (13.5%)
BMI	
<18.5	93 (15.5%)
18.5–24.9	313 (52.16%)
25–29.9	113 (18.83%)
30–34.9	47 (7.83%)
>35	34 (5.67%)
Region of study	
Al-Baha Region	7 (1.16%)
Aseer Region	27 (4.5%)
Eastern Region	60 (10%)
Jizan Region	7 (1.16%)
Madinah Region	127 (21.16%)
Qassim Region	7 (1.16%)
Riyadh Region	365 (60.83%)
Chronic disease	
Yes	53 (8.84%)
No	547 (91.16%)

BMI = body mass index, GPA = grade point average.

Regarding smoking status, 86.5% of participants identified as nonsmokers, 7.83% as former smokers, and 5.67% as current smokers. In terms of living arrangements, 65.5% lived with their families, while 34.5% did not.

When academic performance was assessed by GPA, 33.33% of students reported a GPA below 3.0, 23.33% fell in the 3.0 to 3.5 range, 32.16% reported a GPA between 3.5 and 4.0, 27.66% between 4.0 and 4.5, and 13.5% reported a GPA above 4.5.

BMI classifications revealed that 15.5% of participants were underweight (<18.5), 52.16% had normal weight (18.5–24.9), 18.83% were overweight (25–29.9), 7.83% were in the obese class I category (30–34.9), and 5.67% were classified as obese class II or higher (>35).

Participants represented multiple regions across Saudi Arabia, with the largest proportion from Riyadh Region (60.83%), followed by Madinah Region (21.16%), Eastern Region (10%), and smaller percentages from Aseer (4.5%), Al-Baha (1.16%), Jizan (1.16%), and Qassim (1.16%) regions. The prevalence of self-reported chronic disease was relatively low, with only 8.84% indicating a history of chronic illness (Table 1).

### 3.2. Physical activity

Regarding physical activity, the mean PARS-3 score was 11.16 (SD = 7.41). Most students (88.83%) fell into the low physical activity category (RAS-3 score ≤ 19), while 11.17% were classified as having moderate physical activity (RAS-3 score of 20–42). Notably, no participants met the threshold for high physical activity (RAS-3 score ≥ 43) (Table 2).

**Table 2:** Physical activity (PARS-3), anxiety symptoms (GAD-3), and depressive symptoms (PHQ-9) of the study participants.

Scale	Value (N = 600)
PARS-3 score (mean ± SD)	11.16 ± 7.41
PARS-3 category	
Low (≤19)	533 (88.83%)
Moderate (20–42)	67 (11.17%)
High (≥43)	0 (0%)
GAD-7 score (mean ± SD)	4.5 ± 4.30
GAD-7 category	
Minimal (0–4)	346 (57.66%)
Mild (5–9)	186 (31%)
Moderate (10–14)	47 (7.83%)
Severe (15–21)	20 (3.33%)
PHQ-9 score (mean ± SD)	6.87 ± 4.81
PHQ-9 category	
Minimal (0–4)	221 (36.83%)
Mild (5–9)	253 (42.16%)
Moderate (10–14)	74 (12.33%)
Moderately severe (15–19)	40 (6.66%)
Severe (20–27)	12 (2%)

GAD-7 = Generalized anxiety disorder 7-item scale, PARS-3 = Physical activity range score-3, PHQ-9 = Patient health questionnaire 9-item, SD = Standard deviation.

### 3.3. Anxiety symptoms

Anxiety symptoms assessed with the GAD-7 yielded a mean score of 4.5 (SD = 4.30). Categorically, 57.66% of students reported minimal anxiety (scores 0–4), 31% had mild anxiety (5–9), 7.83% reported moderate anxiety (10–14), and 3.33% were classified as having severe anxiety (15–21) (Table 2).

### 3.4. Depressive symptoms

Depressive symptoms measured with PHQ-9 produced a mean score of 6.87 (SD = 4.81). Distribution across severity categories indicated that 36.83% of students had minimal depressive symptoms (0–4), 42.16% reported mild symptoms (5–9), 12.33% moderate symptoms (10–14), 6.66% moderately severe symptoms (15–19), and 2% severe depressive symptoms (20–27) (Table 2).

### 3.5. Associations between physical activity, anxiety, depression, and academic performance

Associations between categorical classifications of physical activity (PARS-3), anxiety symptoms (GAD-7), depressive symptoms (PHQ-9), and academic performance (GPA categories) were assessed using Pearson chi-square tests. The analysis indicated no statistically significant association between PARS-3 categories and GPA (χ² = 5.89, *P* = .21). Similarly, there was no significant association between GAD-7 severity categories and GPA (χ² = 9.93, *P* = .62), nor between PHQ-9 severity categories and GPA (χ² = 16.75, *P* = .40).

**Figure 1.**
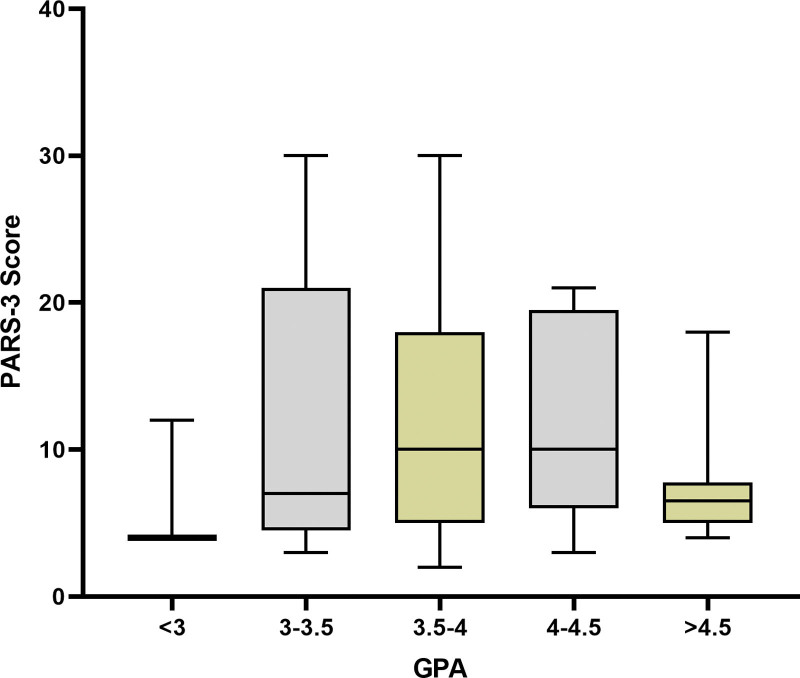
PARS-3 score of the subjects based on GPA categories. GPA = grade point average, PARS-3 = Physical activity range score-3.

For physical activity, students with GPA < 3.0 had a mean PARS-3 score of 6.6 ± 3.7, those with GPA 3.0–3.5 scored 11.8 ± 8.6, GPA 3.5–4.0 scored 11.7 ± 7.6, GPA 4.0–4.5 scored 12.1 ± 6.8, and those with GPA over 4.5 scored 7.9 ± 4.6. The ANOVA test indicated no significant difference in PARS-3 scores among the GPA groups (Fig. 1, *P* = .40).

**Figure 2.**
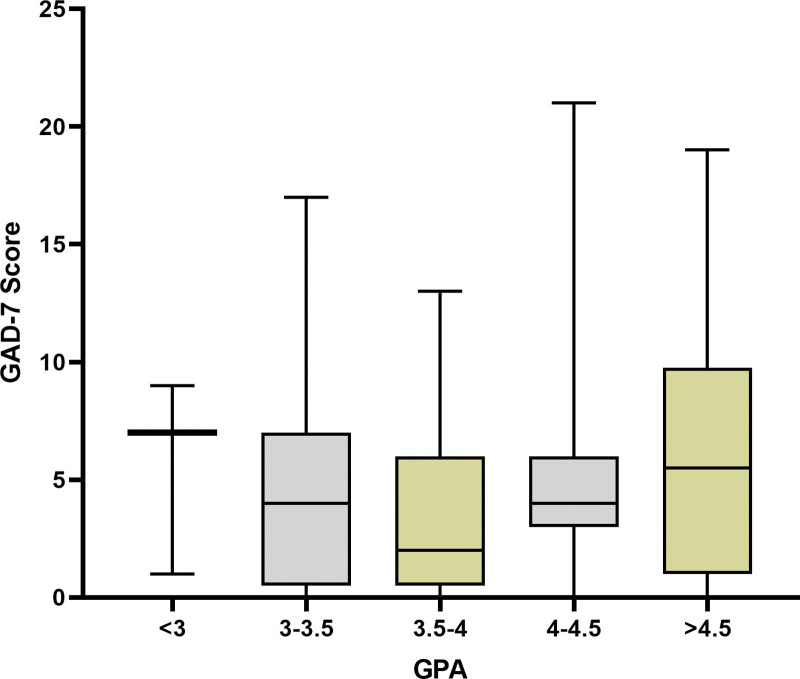
GAD-7 score of the subjects based on GPA categories. GPA = grade point average, GAD-7 = Generalized anxiety disorder 7-item scale.

For anxiety symptoms (GAD-7), students with GPA < 3.0 had a mean score of 5.6 ± 3.4, GPA 3.0–3.5 scored 4.4 ± 4.3, GPA 3.5–4.0 scored 3.4 ± 3.7, GPA 4.0–4.5 scored 4.8 ± 3.8, and GPA over 4.5 scored 6.2 ± 5.6. Also, the ANOVA test revealed no significant differences in GAD-7 scores among GPA categories (Fig. 2, *P* = .41).

**Figure 3.**
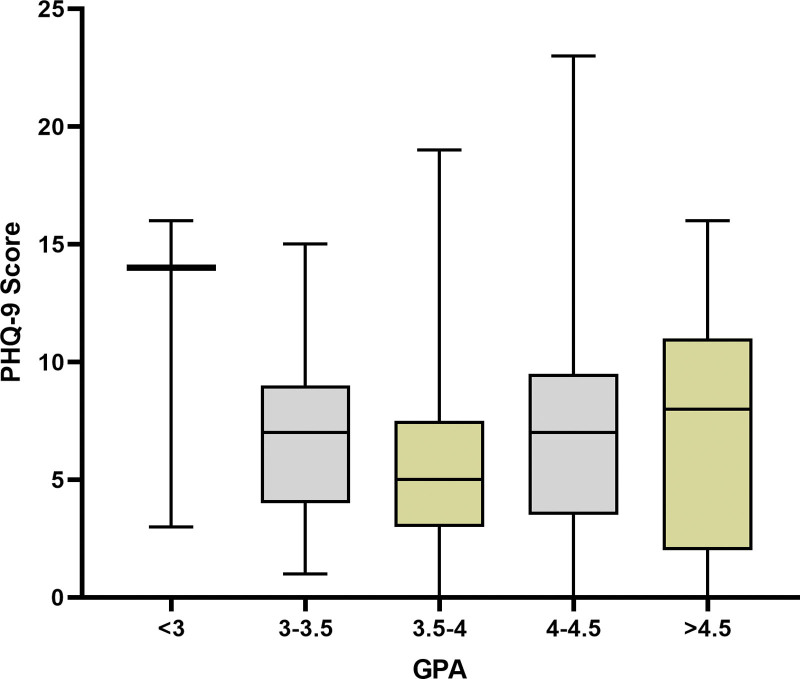
PHQ-9 score of the subjects based on GPA categories. GPA = grade point average, PHQ-9 = Patient health questionnaire 9-item.

For depressive symptoms (PHQ-9), mean scores were 11 ± 5.7 for GPA < 3.0, 7.2 ± 3.9 for GPA 3.0–3.5, 5.5 ± 4.1 for GPA 3.5–4.0, 7.6 ± 5.5 for GPA 4.0–4.5, and 7.3 ± 5.0 for GPA over 4.5. The ANOVA test again indicated no significant differences in PHQ-9 scores across GPA groups (Fig. 3, *P* = .26).

### 3.6. Multivariate regression

A multivariable logistic regression was performed to assess the independent associations of physical activity, anxiety, and depressive symptoms with academic performance, while adjusting for potential confounding demographic factors. The results did not indicate that PARS-3, GAD-7, and PHQ-9 scores were independent predictors of academic performance (Table 3). However, it was observed that higher age groups were associated with decreased odds of high GPA (OR = 0.19, 95% CI: 0.03 to 0.97, *P* = .046). On the other hand, living with one’s family was significantly associated with increased odds of high GPA (OR = 4.64, 95% CI: 1.55 to 13.89, *P* = .006).

**Table 3:** Multivariate logistic regression to identify the independent predictors of academic performance.

Variable	OR	95% CI	*P* value
Gender	0.359	0.9 to 1.31	.12
Age	0.193	0.04 to 0.97	.046
Nationality	0.678	0.03 to 12.17	.79
Marital status	0.116	0.00 to 3.81	.23
Smoking status	0.959	0.24 to 3.74	.95
Living arrangement	4.641	1.55 to 13.89	.006
BMI	0.931	0.56 to 1.52	.77
Region of study	0.949	0.70 to 1.29	.74
Chronic diseases	1.256	0.18 to 8.45	.81
PARS-3 score	0.993	0.91 to 1.07	.87
GAD-7 score	1.116	0.91 to 1.35	.27
PHQ-9 score	0.965	0.82 to 1.13	.66

BMI = Body mass index, CI = Confidence interval, GAD-7 = Generalized anxiety disorder 7-item scale, OR = Odds ratio, PARS-3 = Physical activity range score-3, PHQ-9 = Patient health questionnaire 9-item.

## 4. Discussion

This study investigated plausible associations between physical activity, mental health symptoms, and academic performance among physical therapy students in Saudi Arabia. While the descriptive findings revealed a high prevalence of low physical activity and notable rates of mental health symptoms among participants, our analyses did not identify significant associations between these variables and academic performance as measured by GPA.

The majority of the students (88.83%) were classified in the low physical activity category based on the PARS-3 scale, with no participants meeting the criteria for high physical activity. This aligns with broader evidence from Saudi Arabia indicating suboptimal physical activity levels among university students.^[20]^ Previous research has highlighted multiple barriers to exercise in this context, including academic workload, cultural norms, limited access to facilities, and climatic conditions.^[20]^ Low activity levels among future health professionals are particularly concerning, given their expected role in promoting physical activity among patients. These findings highlight the need for universities to prioritize strategies that facilitate regular physical activity among health-professional students, such as integrating exercise promotion into curricula or providing accessible recreational facilities.

Despite these low levels of physical activity, our results did not demonstrate a statistically significant association between PARS-3 scores and academic performance. This finding contrasts with some international studies reporting a positive link between physical activity and academic achievement, and which propose mechanisms such as improved cognitive function, stress management, and better sleep quality.^[21–23]^ One possible explanation for the lack of any significant association in our sample is that GPA may be influenced by a broader array of factors (including prior academic preparation, teaching quality, study habits, and assessment methods) that could overshadow the effects of physical activity. Additionally, the overwhelmingly low variability in physical activity scores (with nearly 90% classified as low) may have limited our ability to detect meaningful differences across GPA groups.

Similarly, although anxiety and depressive symptoms were common in our sample (with nearly 43% of students reporting mild depressive symptoms and 31% reporting mild anxiety), the study did not find significant associations between GAD-7 or PHQ-9 scores and GPA. This result is somewhat unexpected, given that previous research has consistently shown that psychological distress can negatively impact on academic performance through mechanisms such as reduced concentration, diminished motivation, and impaired memory.^[24–26]^ Several explanations may account for this discrepancy. First, students may have adopted coping strategies that allowed them to maintain academic performance despite experiencing psychological symptoms. Second, GPA as a self-reported and cumulative measure may not capture short-term fluctuations in academic functioning related to acute episodes of anxiety or depression. Third, cultural factors, such as strong familial expectations or social support networks, might buffer the academic impact of mental health symptoms in this population.

Importantly, the descriptive data, nevertheless, point to a significant mental health burden among Saudi physical therapy students. Over 42% of participants reported mild depressive symptoms, while nearly 12% reported moderate or higher levels. Anxiety symptoms were also prevalent, with nearly 11% reporting moderate or severe levels. These figures are consistent with previous studies both in Saudi Arabia and globally that have documented elevated rates of psychological distress among health-professional students.^[27–29]^ The demanding nature of physical therapy programs, which combine academic rigor with clinical training and professional socialization pressures, may contribute to this elevated risk.

Although this study did not demonstrate significant associations between physical activity, mental health symptoms, and academic performance, the high prevalence of low physical activity and psychological distress remains a concern from a student well-being perspective. Interventions to promote mental health and physical activity remain essential for this population, even if they do not directly improve GPA. Universities should consider implementing targeted mental health services, stress management programs, and physical activity promotion initiatives tailored to student needs.

The results of the multivariable logistic regression analysis provide important insights into the factors associated with academic performance among physical therapy students. While physical activity (PARS-3), anxiety (GAD-7), and depressive symptoms (PHQ-9) were not identified as independent predictors of one’s GPA category after adjusting for demographic factors, certain demographic variables showed significant associations. Specifically, students in higher age groups had significantly reduced odds of achieving a high GPA (OR = 0.19), suggesting that older students may face additional challenges of balancing academic demands or may differ in previous academic preparation. Conversely, living with one’s family was strongly associated with increased odds of high GPA (OR = 4.64), which may reflect the positive role of family support, stability, and reduced financial or social stressors in promoting academic success. These findings highlight that while health-related behaviors and mental health symptoms are important to be monitored, demographic and social-context factors can play a critical role in shaping academic outcomes and should be considered when designing interventions to support student performance.

This study has several strengths, including its large sample size, use of validated assessment tools (PARS-3, GAD-7, PHQ-9), and the inclusion of students from many regions across Saudi Arabia. However, several limitations should also be acknowledged. First, the cross-sectional design precludes causal inferences about the relationships between variables. Second, the reliance on self-reported GPA may cause a reporting bias. However, the use of self-reported GPA is a common practice in educational research when official records are not available. Prior research consistently shows that self-reported GPA is reliable and correlated with official academic records.^[30]^ In this study, the anonymous survey design reduced the risk of social desirability bias and encouraged honest reporting. Moreover, students had routine access to their GPA through online university portals in Saudi Arabia, and there were no incentives or benefits for misreporting. Third, the overwhelmingly low physical activity scores limited the ability of the study to examine the impact of moderate-to-high activity levels. Future research should consider longitudinal designs to assess how changes in physical activity and mental health over time relate to academic outcomes. Finally, it should also be noted that of the total initial potential students recruited, 7 did not sign the consent forms and did not participate in the study. In line with ethical requirements, no data were collected from these individuals, and they were not included in the analysis. Because the study was performed anonymously, it was not possible to monitor the characteristics of non-responders or compare them with participants to gain a vivid insight on the potential differences between those who consented and those who refused to take part.

## 5. Conclusion

In conclusion, this study found that while physical therapy students in Saudi Arabia exhibited high rates of low physical activity and notable levels of mental health symptoms, these factors were not significantly associated with academic performance in this sample. Moreover, regression analysis indicated that being older was associated with lower odds of high academic performance, while living with one’s family significantly increased the odds of achieving a high GPA. These results highlight the importance of considering demographic and social-context factors when developing strategies to support student success. Additionally, the findings highlight a clear need for universities to address student well-being holistically, through strategies that promote physical activity, reduce psychological distress, and support students in managing the demands of professional training.

## Acknowledgments

The author would like to thank the Deanship of Scientific Research at Shaqra University for supporting this work.

## Author contributions

**Conceptualization:** Abdullah Ibrahim Alhusayni.

**Data curation:** Abdullah Ibrahim Alhusayni.

**Formal analysis:** Abdullah Ibrahim Alhusayni.

**Investigation:** Abdullah Ibrahim Alhusayni.

**Methodology:** Abdullah Ibrahim Alhusayni.

**Project administration:** Abdullah Ibrahim Alhusayni.

**Resources:** Abdullah Ibrahim Alhusayni.

**Software:** Abdullah Ibrahim Alhusayni.

**Supervision:** Abdullah Ibrahim Alhusayni.

**Validation:** Abdullah Ibrahim Alhusayni.

**Visualization:** Abdullah Ibrahim Alhusayni.

**Writing – original draft:** Abdullah Ibrahim Alhusayni.

**Writing – review & editing:** Abdullah Ibrahim Alhusayni.
